# Supplementary material for: Risk factors for sacrococcygeal pilonidal sinus: a systematic review and meta-analysis supplemented by genetic causal assessment
Source: Front Surg. 2026 Jan 7;12:1718589. doi: 10.3389/fsurg.2025.1718589 (PMC12819706; doi:10.3389/fsurg.2025.1718589)
Supplement: Supplementary file 2 [file Datasheet2.zip › Supplementary Data 2/MR_pipeline_p5e-6/scatter_plots/ebi-a-GCST005814.finngen_R12_L12_PILONIDALCYST.pdf]

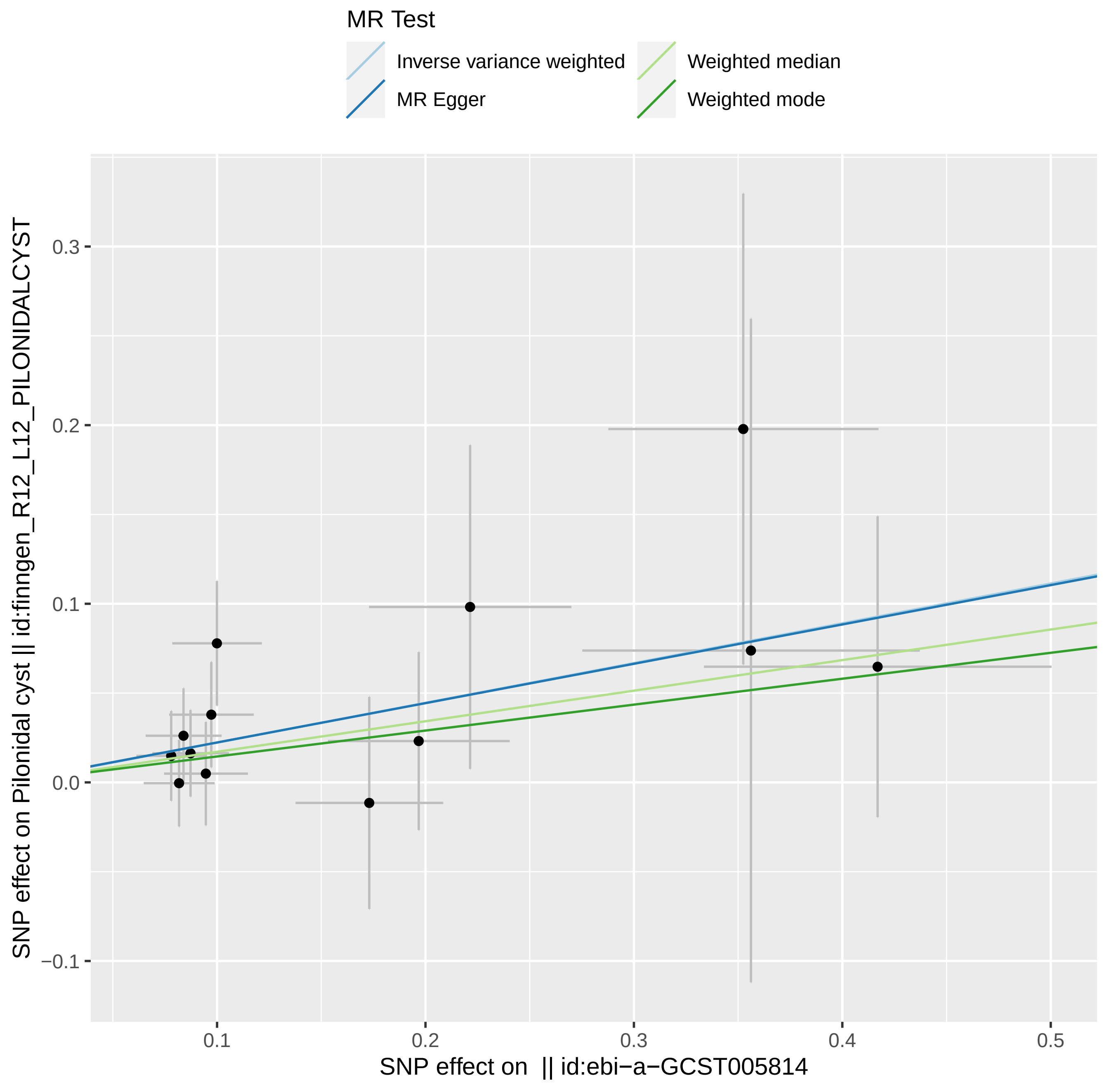
MR Test
Inverse variance weighted
MR Egger
Weighted median
Weighted mode
SNP effect on Pilonidal cyst || id:finngen_R12_L12_PILONIDALCYST
0.3
0.2
0.1
0.0
−0.1
0.1
0.2
0.3
0.4
0.5
SNP effect on || id:ebi−a−GCST005814
